# Use of cardiac magnetic resonance imaging after hospitalization for acute heart failure: insights from the French nationwide OFICA-2 cohort

**DOI:** 10.1093/ehjimp/qyag159

**Published:** 2026-09-10

**Authors:** Alexandre Altes, Benjamin Alos, Damien Legallois, Maxime Doublet, Jerôme Costa, Aymeric Menet, Etienne Puymirat, Alexis Jacquier, Christophe Thuaire, François Picard, Jean-Noel Trochu, Nicolas Lamblin, Clément Delmas, Ariel Cohen, Charlotte Dagrenat, Lise Legrand, Marjorie Canu, Jerôme Corre, Laurent Desprets, Jeremy Laik, Thibaud Genet, Hubert Cochet, Nathan Mewton, Damien Logeart, Claire Bouleti

**Affiliations:** GCS-Groupement des Hôpitaux de l’Institut Catholique de Lille/Lille Catholic Hospitals, Heart Valve Center, Cardiology Department, ETHICS EA 7446, DataCoeur, Lille Catholic University, Lille, France; Cardiology Department, University Hospital of Poitiers, Clinical Investigation Center (INSERM 1402), 2, Rue de la Milétrie, Poitiers 86000, France; Department of Cardiology, Normandie University, UNICAEN, INSERM U1086 ANTICIPE, Caen-Normandy University Hospital, Avenue de la Côte de Nacre, CAEN F-14000, France; Biostatistical Department, Clinityx Company, Boulogne-Billancourt, France; Cardiology Department, Reims University Hospital, Reims, France; GCS-Groupement des Hôpitaux de l’Institut Catholique de Lille/Lille Catholic Hospitals, Heart Valve Center, Cardiology Department, ETHICS EA 7446, DataCoeur, Lille Catholic University, Lille, France; Cardiology Department, European Hospital Georges Pompidou, AP-HP, Paris, France; Radiology Department, University Hospital of Marseille, AP-HM, Marseille, France; Service de cardiologie, Les Hôpitaux de Chartres, Chartres, Centre-Val de Loire, France; Service de traitement de l’insuffisance cardiaque, CHU de Bordeaux, Bordeaux, France; Institut du Thorax, Hôpital Universitaire, Nantes Université, Centre National de Recherche Scientifique, INSERM, Nantes, France; Inserm, Université de Lille, CHU Lille, Institut Pasteur Lille, U1167-RID-AGE-Facteurs de risque et déterminants moléculaires des maladies liées au vieillissement, Lille F-59000, France; Cardiology Department, Toulouse University Hospital, University of Toulouse, Toulouse, France; REICATRA, Institut Saint Jacques, Toulouse, France; Cardiology Department, Saint-Antoine University Hospital, AP-HP, Paris, France; Service de Cardiologie, Centre Hospitalier de Haguenau, Haguenau, France; ACTION Study Group, Sorbonne Université, INSERM UMRS 1166, Institut de Cardiologie, Hôpital Pitié-Salpêtrière (AP-HP), Paris 75013, France; Department of Cardiology, University Hospital, Grenoble Alpes 38000, France; Service de Cardiologie, CHU Felix Guyon, Réunion, France; Department of Cardiology, Cholet Hospital, Cholet, France; Department of Echocardiography, Cardio X Clinic, Cannes, France; Cardiology Department, University Hospital of Tours, Tours, France; Department of Cardiovascular Imaging, CHU Bordeaux IHU Liryc, CHU/Univ. Bordeaux/INSERM 1045, Bordeaux, France; Cardiology Institute of the Hospices Civils de Lyon, CarMeN INSERM 1060 Unit, Claude Bernard, Lyon 1 University, Lyon, France; Inserm U942, Paris Cité University, AP–HP Hôpital Lariboisière, Paris 75010, France; Cardiology Department, University Hospital of Poitiers, Clinical Investigation Center (INSERM 1402), 2, Rue de la Milétrie, Poitiers 86000, France

**Keywords:** acute heart failure, cardiac magnetic resonance imaging, healthcare disparities, guideline adherence

## Abstract

**Aims:**

Cardiac magnetic resonance (CMR) has a central role in the management of patients with acute heart failure (AHF), but timely access remains heterogeneous. We investigated real-world CMR use in patients hospitalized for AHF and its associations with demographic, clinical, and institutional characteristics.

**Methods and results:**

In the nationwide prospective OFICA2 cohort, conducted from March to April 2021 across 80 centres, we included 906 patients hospitalized for AHF, discharged alive, and without contraindications to CMR (mean age 74.7 years, 42.1% women). CMR use was identified through national billing codes during hospitalization and within 1 year using linked national administrative data. Analyses assessed CMR use across patient subgroups, hospital types, and care settings. Overall, 116 patients (12.8%) underwent CMR during hospitalization or within 1 year. CMR use was more frequent in younger patients, those without myocardial infarction, those with reduced LVEF, and patients with dilated rather than hypertensive or valvular cardiomyopathy. Among patients hospitalized for a first AHF episode, male sex was also associated with higher referral rates. CMR use varied across hospital types and care settings, with higher rates in university hospitals and intensive care units. Among patients with potential CMR indications according to the 2016 ESC Heart Failure guidelines, only 10.9% (class I) and 15.0% (class II) underwent the examination.

**Conclusion:**

In this nationwide AHF cohort, real-world use of CMR remained limited, even among patients with potential guideline-based indications, with marked patient- and institution-level disparities. These findings support coordinated efforts to improve timely access to CMR in heart failure care.

## Graphical Abstract

**Graphical Abstract:**
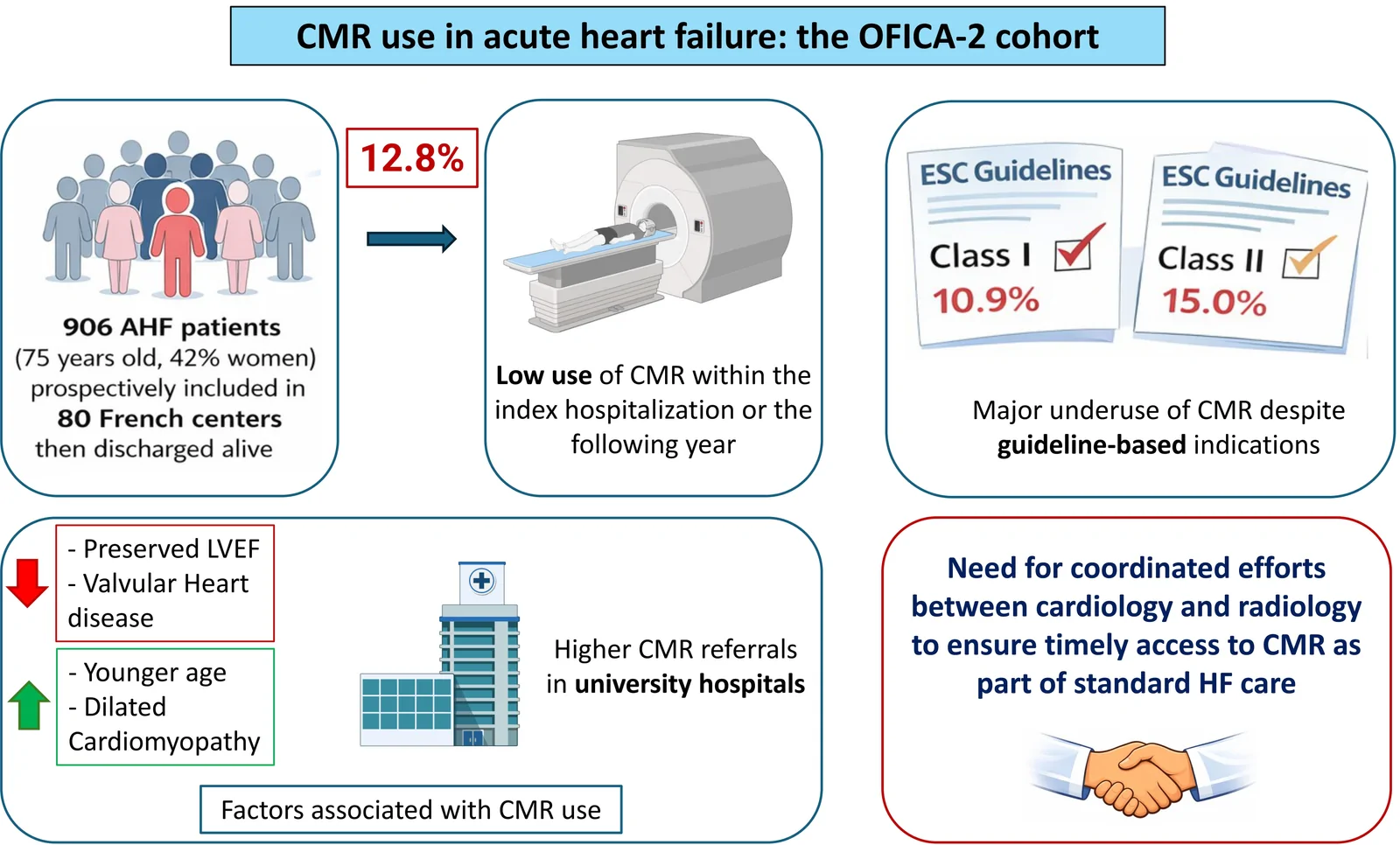
Underuse of CMR in patients hospitalized for acute heart failure. Among 906 patients included across 80 centres, only 12.8% underwent CMR during the index hospitalization or within 1 year. CMR use was more frequent in younger patients, those with dilated cardiomyopathy, reduced LVEF, and in university hospitals. Concordance with the 2016 ESC guideline-based indications was low. AHF, acute heart failure; CMR, cardiac magnetic resonance; ESC, European Society of Cardiology; LVEF, left ventricular ejection fraction.

## Introduction

Acute heart failure (AHF) remains a major cardiovascular emergency associated with a substantial socio-economic burden.^1^ Accurate identification of its underlying causes relies on a multimodal approach combining clinical, biological, and imaging data. While echocardiography remains the first-line imaging modality in AHF, cardiac magnetic resonance (CMR) has become the reference non-invasive method for assessing cardiac chamber remodelling and systolic function. Additionally, CMR provides advanced myocardial tissue characterization, allowing more precise identification of AHF aetiologies.^2^ CMR also contributes to risk stratification, device-therapy selection in HF with reduced ejection fraction (HFrEF), and monitoring of disease progression or treatment response in several cardiomyopathies.^3^

Worldwide, CMR referrals have increased considerably.^4^ CMR is now integrated into multiple practice guidelines, including those for ischaemic heart disease, cardiomyopathies, and valvular heart diseases.^5^ However, access remains highly variable and insufficient. Contributing factors include limited scanner availability, long waiting times, a shortage of physicians and technologists trained in CMR, heterogeneous clinical practice between centres, and inadequate compensation relative to the long duration of the examination.

Recent survey-based data have highlighted substantial barriers to CMR delivery across Europe.^6^ However, patient-level data on actual CMR use after hospitalization for AHF, and on the clinical factors associated with its use, remain scarce. In this context, the OFICA2 cohort provides an opportunity to assess current patterns of CMR use in patients hospitalized for AHF. The primary objective of this study was to describe contemporary CMR use in this population. Secondary objectives were to identify the demographic, clinical, and institutional factors associated with CMR examinations.

## Methods

The design and main results of the OFICA2 cohort have been reported previously.^7,8^ Briefly, OFICA2 is a national, prospective, multicentre, observational cohort of patients with AHF requiring hospitalization, regardless of left ventricular ejection fraction (LVEF) or hospital ward, and presenting with chronic or *de novo* HF.

### Study population

A total of 80 centres participated in the study (31 university hospitals, 42 community hospitals, and 7 private centres). Investigators were asked to include all patients hospitalized for AHF during three consecutive weeks between March and April 2021, no matter the unit of admission. Inclusion criteria were patients aged >18 years, affiliation with the French health insurance, and hospitalization for suspicion of AHF based on the definition of ESC guidelines. Exclusion criteria were a final diagnosis of AHF not retained or refusal to participate. There was no pre-determined target number of inclusions. The study size was determined by the number of consecutive eligible patients enrolled in the OFICA2 cohort during the predefined inclusion period.

For the present ancillary study, patients who died during the index hospitalization and those with a contraindication to CMR were also excluded. Contraindications to CMR were pacemaker or implantable cardioverter-defibrillator (ICD), chronic dialysis, or an estimated glomerular filtration rate (eGFR) < 30 mL/min. The presence of a pacemaker or ICD is not an absolute contraindication to CMR when MRI-conditional devices are used. However, this information could not be reliably assessed, and device-related artefacts expected in patients with pacemakers/ICDs justified their exclusion from the present study.

### Data collection

Data were extracted from the OFICA2 cohort, which integrates a detailed case report form (CRF) completed during the index hospitalization with linkage to the *Système National des Données de Santé* (SNDS; French National Health Data System) for follow-up. The SNDS provided imaging examinations during follow-up and in the 2 years preceding the index AHF hospitalization. The primary variable of interest was the utilization of CMR, identified through national billing codes. The prevalence of CMR examinations was assessed during the index hospitalization and within the following year. This 1-year window was selected to capture not only CMR performed during the acute hospitalization but also examinations deferred until clinical stabilization and outpatient reassessment because of the patient’s acute condition, limited immediate access to CMR, or the need to reschedule an initially planned examination. We also reported CMR use in the 2 years preceding hospitalization, as some patients with pre-existing cardiac disease may have undergone CMR for diagnostic purposes before admission.

### Study endpoints

The primary endpoint was the proportion of patients who underwent CMR either during the index hospitalization for AHF or within 1 year following discharge, among the whole population and those without a previous history of heart failure hospitalization or known structural heart disease. CMR examinations in the 2 years preceding index hospitalization were not part of the endpoint.

Secondary endpoints included the following:

- Clinical and demographic factors independently associated with CMR use.
- CMR examination rates according to the underlying cardiac disease.
- Comparison of CMR rates across hospital types and wards.
- Median time (in days) between the index admission for AHF and CMR.
- Proportion of patients who underwent CMR as recommended by the 2016 ESC Guidelines for the Diagnosis and Treatment of Acute and Chronic Heart Failure (class I and II indications).^9^

### Ethical and regulatory considerations

OFICA2 was conducted in accordance with the ethical principles of the Declaration of Helsinki, the International Conference on Harmonization/Good Clinical Practice, and applicable regulatory requirements (IRB n° 19.12.12.63430, ID RCB: 2019-A03222-55 and CNIL n° 919 234). The survey was registered on *Clinicaltrials.gov* under NCT05232058. This manuscript complies with the STROBE statement.

### Statistical analysis

Continuous variables were expressed as median (interquartile range, IQR) or mean and standard deviation and compared using Student’s *t*-test or Wilcoxon Rank Sum test, depending on the distribution. Discrete variables were expressed as numbers (percentage) and compared using Pearson’s chi-square test with Yates’ continuity correction or Fisher’s exact test. A multivariable logistic regression model was constructed to identify factors independently associated with CMR use. Candidate variables were selected based on their univariable association with CMR use and their clinical relevance. No automated variable-selection procedure, such as stepwise, forward, or backward selection, was used. Prior to model fitting, potential multicollinearity was assessed by examining the correlation matrix of all candidate variables and calculating variance inflation factors. Variables showing excessive collinearity or extremely low prevalence were reviewed and excluded when appropriate.

The final model was estimated using standard maximum likelihood logistic regression. Only complete cases were included, as the proportion of missing data was minimal and no imputation was performed. Model convergence was verified, and the standard errors of the estimated coefficients were examined to ensure numerical stability and to rule out issues related to sparse data or quasi-separation. Odds ratios (ORs) and their 95% confidence intervals were obtained by exponentiating the model coefficients. Analyses were performed in the overall study population and among patients hospitalized for a first episode of AHF, pre-specified as a subgroup. Potential sources of bias were addressed through consecutive patient inclusion during a predefined period, standardized data collection, and multivariable adjustment for confounding. A *P*-value <0.05 (two-tailed test) was considered statistically significant. All analyses were performed using Python in a secure environment.

## Results

### Study population

**Figure 1:**
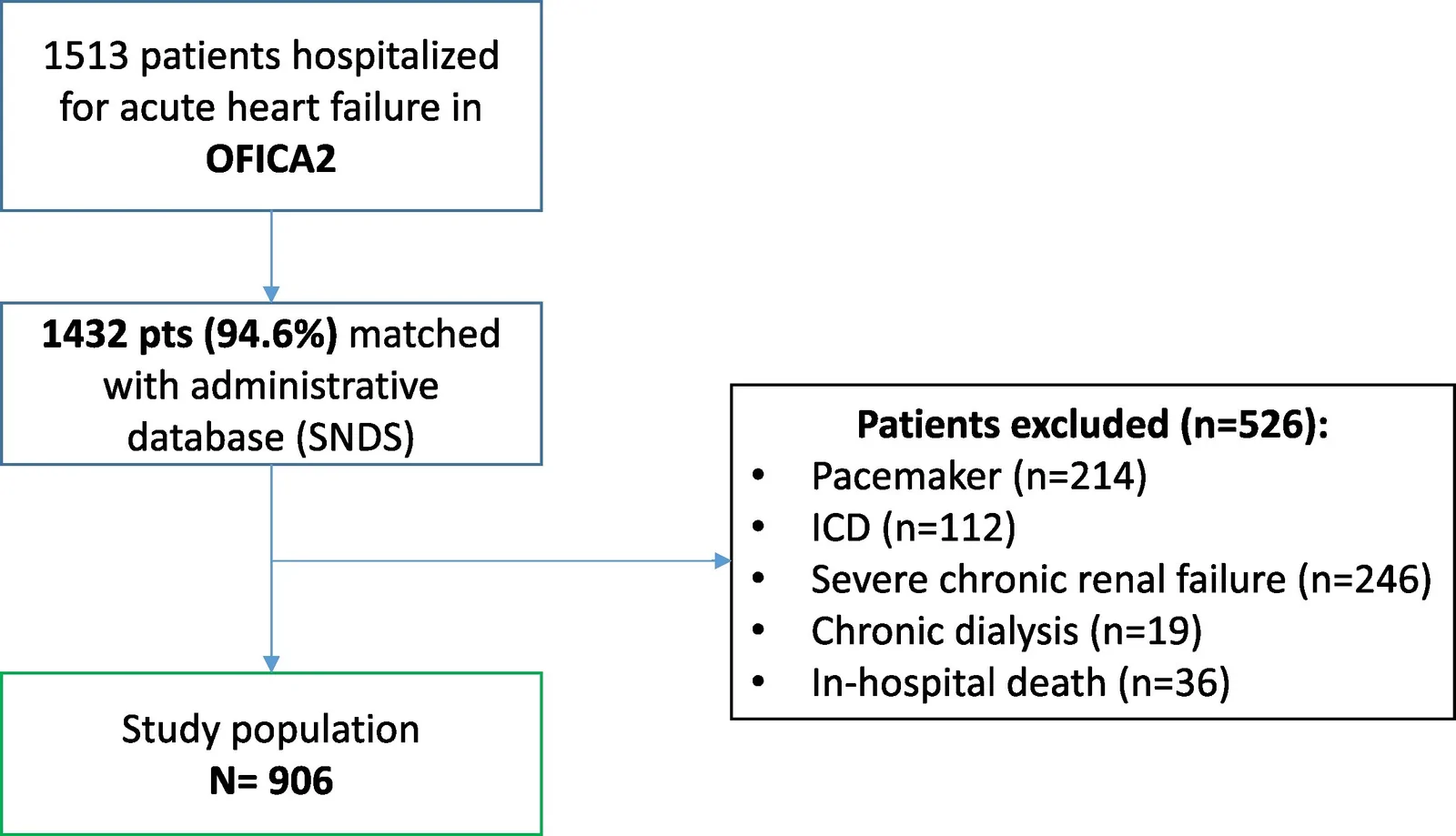
Flow chart of the study population. ICD, implantable cardioverter defibrillator; SNDS, *Système National des Données de Santé.*

Of the 1513 patients enrolled in OFICA2, 1432 were successfully matched with the SNDS database. After excluding patients who died during the index hospitalization or had contraindications to CMR acquisition (pacemaker/ICD, chronic dialysis, eGFR <30 mL/min) (*Figure 1*), 906 patients constituted the final study population (mean age 74.7 ± 14.1 years, 42.4% women).

### CMR use after hospitalization for AHF

Among the 906 patients included, 116 (12.8%) underwent CMR either during the index hospitalization (*n* = 62, 6.8%) or within the subsequent year (*n* = 64, 7.1%, including 10 patients who had already undergone CMR during the initial admission). In addition, 31 patients (3.4%) had undergone CMR in the 2 years preceding admission. In total, 147 patients (16.2%) had at least one CMR, including 7 who underwent the examination both before and after the index hospitalization (see Supplementary data online, *Tables S1* and *S2*).

When CMR was performed during the index admission, the median time to the examination was 6.0 days (IQR 4.25–11). When performed within the year following discharge, the median delay was 71.5 days (IQR 37.25–162.5), and 46 patients (39.7%) had the CMR within the first month.

Patients who underwent CMR were younger and more frequently men (all *P* ≤ 0.032, *Table 1*). They were less often hypertensive, more frequently active smokers, and more commonly in sinus rhythm (all *P* < 0.001). They had undergone fewer PCIs, had less cerebrovascular disease, and showed higher haemoglobin and eGFR values at admission (all *P* ≤ 0.020).

**Table 1:** Baseline clinical and demographic characteristics by CMR use

Variables	Patients eligible to CMR *N* = 906	Patients with CMR *N* = 116 (12.8%)	Patients without CMR *N* = 790 (87.2%)	Overall *P*-value	Patients with available data, n
**Demographics**					
Age (years)	74.7 ± 14.1	61.5 ± 16.1	76.7 ± 12.7	**<0**.**001**	906
Male gender	522 (57.6%)	78 (67.2%)	444 (56.2%)	**0**.**032**	906
Hypertension	614 (68.1%)	55 (47.4%)	559 (71.1%)	**<0**.**001**	902
Active smoker	133 (14.8%)	31 (26.7%)	102 (13.1%)	**<0**.**001**	896
Diabetes mellitus	316 (35.2%)	34 (29.3%)	282 (36.1%)	0.185	898
SBP (mmHg)	136.0 ± 28.3	135.4 ± 29.0	136.1 ± 28.2	0.781	891
Heart rate (b.p.m.)	92.8 ± 26.2	97.3 ± 26.3	92.2 ± 26.1	0.051	894
Sinus rhythm	480 (53.6%)	81 (70.4%)	399 (51.2%)	**<0**.**001**	895
BMI (kg/m^2^)	28.5 ± 7.3	27.6 ± 6.7	28.7 ± 7.3	0.156	796
Obesity	263 (33.0%)	26 (24.8%)	237 (34.3%)	0.068	796
**Cardiovascular history**					
Past HF hospitalization	333 (37.1%)	34 (29.3%)	299 (38.2%)	0.079	898
MI	163 (18.1%)	13 (11.2%)	150 (19.1%)	0.054	903
PCI	166 (18.4%)	10 (8.6%)	156 (19.8%)	**0**.**005**	903
CABG	62 (6.9%)	6 (5.2%)	56 (7.1%)	0.565	903
Valvular surgery	60 (6.7%)	3 (2.6%)	57 (7.3%)	0.091	901
TAVI	25 (2.8%)	0 (0%)	25 (3.2%)	0.100	902
Cerebrovascular disease	89 (9.9%)	4 (3.4%)	85 (10.8%)	**0**.**020**	902
**Non cardiovascular history**					
Alcohol abuse	90 (10.0%)	18 (15.5%)	72 (9.2%)	0.052	898
Treated obstructive sleep apnoea	85 (9.4%)	11 (9.5%)	74 (9.4%)	1	903
Treated COPD	159 (17.6%)	20 (17.2%)	139 (17.7%)	1	901
Treated depression	78 (8.6%)	6 (5.2%)	72 (9.1%)	0.221	902
Cirrhosis	34 (3.8%)	2 (1.7%)	32 (4.1%)	0.325	899
Active or recent (<5 years) cancer	74 (8.2%)	8 (6.9%)	66 (8.4%)	0.707	900
Severe cognitive impairment	37 (4.1%)	1 (0.9%)	36 (4.6%)	0.105	901
Charlson comorbidity index	3.8 ± 3.2	3.3 ± 3.0	3.8 ± 3.3	0.118	906
**Biology**					
Haemoglobin (g/dL)	12.4 ± 2.3	13.2 ± 2.3	12.3 ± 2.3	**<0**.**001**	867
eGFR (mL/min/1.73 m^2^)	65.1 ± 21.9	77.6 ± 24.4	63.3 ± 21.0	**<0**.**001**	880
**Index hospitalization**					
Type of hospital					906
University hospital	532 (58.7%)	92 (79.3%)	440 (55.7%)	**<0**.**001**	
General hospital	288 (31.8%)	20 (17.2%)	268 (33.9%)	**<0**.**001**	
Private hospital	86 (9.5%)	4 (3.4%)	82 (10.4%)	**0**.**027**	
Hospitalization wards					906
Cardiology wards	502 (55.4%)	55 (47.4%)	447 (56.6%)	0.079	
ICCU/ICU	258 (28.5%)	54 (46.6%)	204 (25.8%)	**<0**.**001**	
Geriatry wards	59 (6.5%)	1 (0.9%)	58 (7.3%)	**0**.**015**	
Medicine wards	78 (8.6%)	5 (4.3%)	73 (9.2%)	0.112	
Other	1 (0.1%)	0 (0%)	1 (0.1%)	1	

*P* values in bold are below the 0.05 threshold.

BMI, body mass index; CABG, coronary artery bypass grafting; COPD, chronic obstructive pulmonary disease; eGFR, estimated glomerular filtration rate; HF, heart failure; ICCU, intensive cardiac care unit; ICU, intensive care unit; MI, myocardial infarction; PCI, percutaneous coronary intervention; SBP, systolic blood pressure; TAVI, transcatheter aortic valve implantation.

Among the 461 patients admitted for a first episode of AHF without known structural or ischaemic heart disease (see Supplementary data online, *Tables S3* and *S4*), 76 (16.5%) underwent CMR during hospitalization or within 1 year after discharge.

### Rates of CMR examinations according to cardiac disease

**Figure 2:**
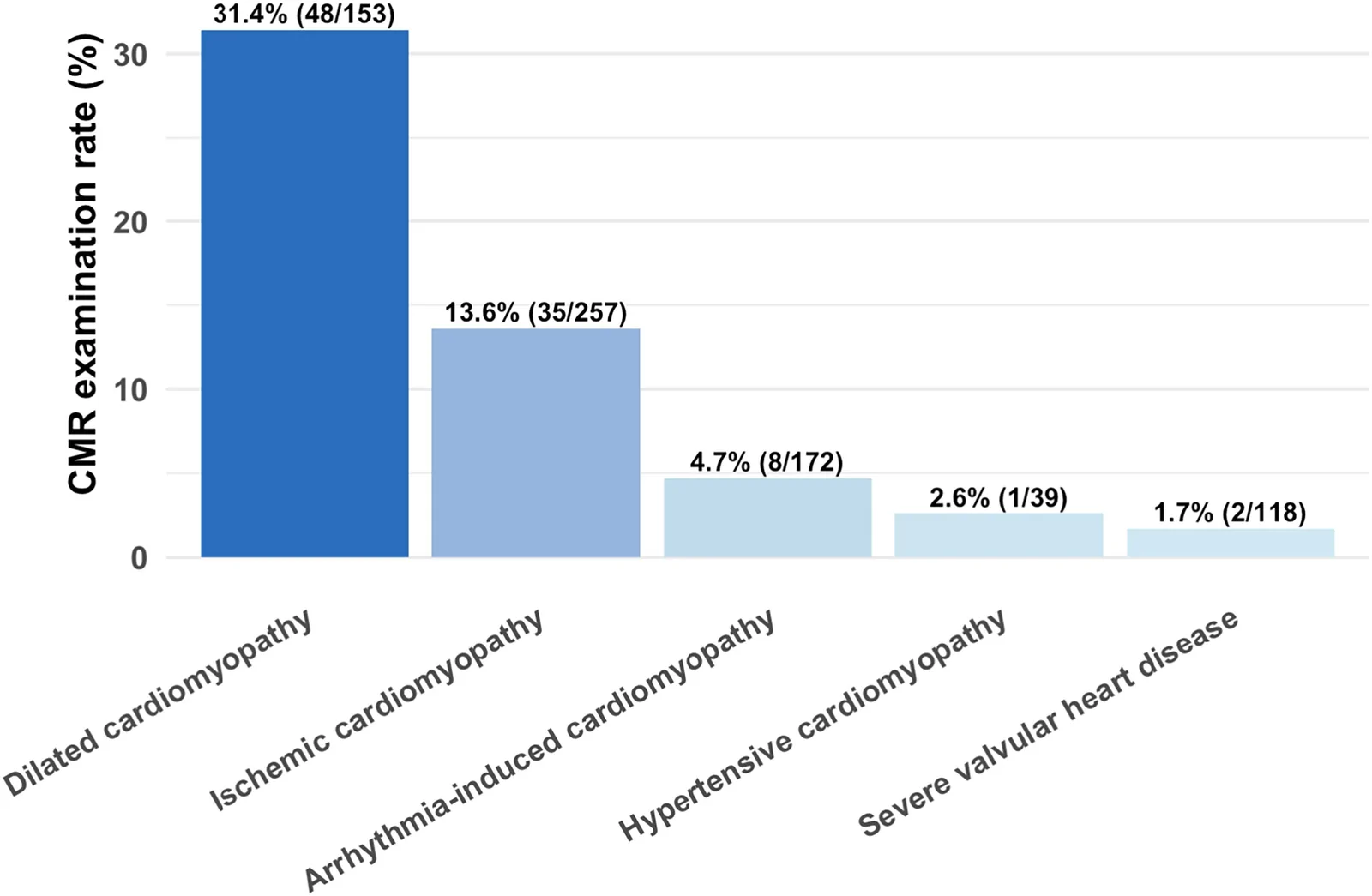
Rates of CMR according to underlying cardiac disease.

CMR rates varied substantially according to the underlying cardiac disease (*P* < 0.001, Supplementary data online, *Table S5*, *Figure 2*). The highest rates were seen in myocarditis (*n* = 3/4, 75.0%, interpreted cautiously due to small numbers) and in dilated cardiomyopathy (*n* = 48/153, 31.4%), followed by ischaemic cardiomyopathy (*n* = 35/257, 13.6%). In contrast, CMR was infrequent in arrhythmia-induced cardiomyopathy (*n* = 8/172, 4.7%), hypertensive disease (*n* = 1/39, 2.6%), and severe valvular heart disease (*n* = 2/118, 1.7%).

### Factors independently associated with CMR use

**Figure 3:**
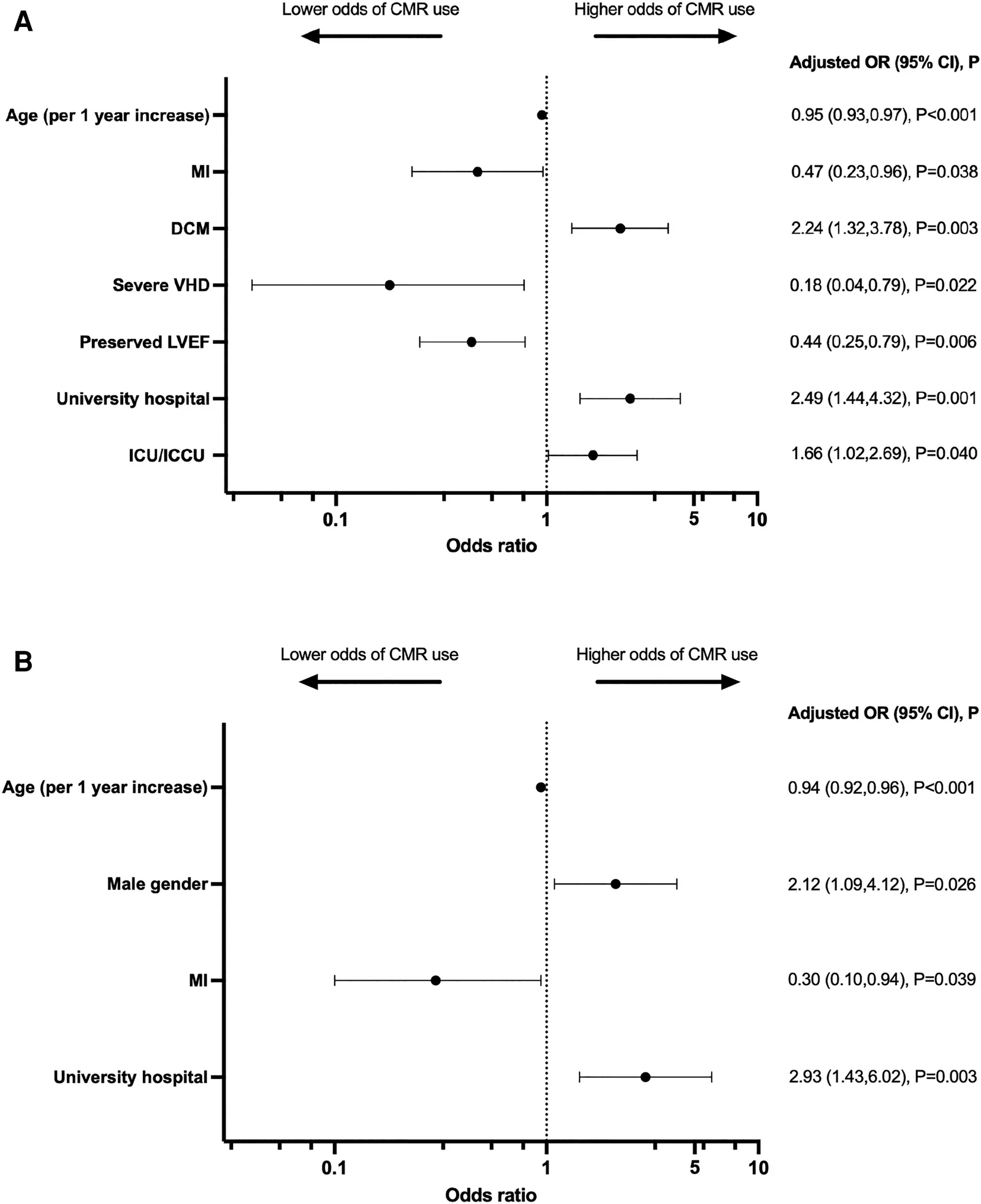
Forest plot of independent factors associated with CMR use for the whole cohort (*A*) or in patients hospitalized for first episode of acute heart failure (*B*). DCM, dilated cardiomyopathy; ICCU, intensive cardiac care unit; ICU, intensive care unit; LVEF, left ventricular ejection fraction; MI, myocardial infarction; VHD, valvular heart disease.

Univariate and multivariable analyses of factors associated with CMR examinations are shown in *Table 2*. On multivariable analysis, younger age, absence of myocardial infarction (MI), admission to a university hospital, admission to an ICCU/ICU, an initial diagnosis of dilated cardiomyopathy, absence of severe valvular heart disease, and reduced LVEF were independently associated with higher odds of CMR examination. (*Figure 3A*).

**Table 2:** Factors associated with CMR examination, either during the index hospitalization for AHF or within the following year, on univariate and multivariable analysis

	Univariate analysis			Multivariable analysis (*n* = 860)	
Covariates	OR (95% CI)	*P*-value	Patients with available data, n	OR (95% CI)	*P-*value
Male gender	1.60 (1.06–2.42)	0.026	906	1.54 (0.89–2.65)	0.122
Age (year)	0.93 (0.92–0.95)	<0.001	906	0.95 (0.93–0.97)	**<0**.**001**
Hypertension	0.37 (0.25–0.54)	<0.001	902		
Active smoker	2.42 (1.53–3.84)	<0.001	896		
Diabetes mellitus	0.68 (0.46–1.00)	0.053	897		
Heart rate (b.p.m)	1.01 (1.00–1.01)	0.052	894	1.00 (0.99–1.01)	0.430
Sinus rhythm	2.27 (1.49–3.48)	<0.001	895	1.38 (0.82–2.34)	0.225
Obesity	0.63 (0.39–1.01)	0.054	796		
MI	0.54 (0.29–0.98)	0.043	903	0.47 (0.23–0.96)	**0**.**038**
PCI	0.38 (0.19–0.75)	0.005	903		
Valvular surgery	0.34 (0.10–1.10)	0.072	901		
Cerebrovascular disease	0.29 (0.11–0.82)	0.019	902		
Alcohol abuse	1.81 (1.04–3.16)	0.037	898		
Severe cognitive impairment	0.18 (0.02–1.35)	0.095	901		
Haemoglobin (g/dL)	1.20 (1.09–1.32)	<0.001	867		
eGFR (mL/min/1.73m^2^)	0.99 (0.99–1.00)	0.041	880	0.99 (0.99–1.00)	0.125
University hospital	2.80 (1.69–4.65)	<0.001	906	2.49 (1.44–4.32)	**0**.**001**
Private hospital	0.65 (0.22–1.97)	0.449	906		
Cardiology wards	0.69 (0.47–1.02)	0.065	906		
ICCU/ICU	2.50 (1.68–3.73)	<0.001	906	1.66 (1.02–2.69)	**0**.**040**
DCM	4.05 (2.11–7.75)	<0.001	881	2.24 (1.32–3.78)	**0**.**003**
Arrhythmia-related	0.43 (0.18–1.06)	0.067	881		
Hypertensive	0.23 (0.03–1.83)	0.166	881		
Ischaemic	1.40 (0.72–2.70)	0.320	881		
Myocarditis	26.57 (2.59–273.01)	0.006	881		
Severe VHD	0.15 (0.03–0.69)	0.014	881	0.18 (0.04–0.79)	**0**.**022**
Reduced LVEF (<40%)	4.22 (2.75–6.48)	<0.001	906		
Preserved LVEF (>50%)	0.28 (0.17–0.45)	<0.001	906	0.44 (0.25–0.79)	**0**.**006**
Left-sided HF	1.62 (1.09–2.39)	0.016	906		
Global HF	0.75 (0.47–1.22)	0.246	906		

*P* values in bold are below the 0.05 threshold.

BMI, body mass index; CABG, coronary artery bypass grafting; COPD, chronic obstructive pulmonary disease; DCM, dilated cardiomyopathy; eGFR, estimated glomerular filtration rate; HF, heart failure; ICCU, intensive cardiac care unit; ICU, intensive care unit; MI, myocardial infarction; PCI, percutaneous coronary intervention; SBP, systolic blood pressure; TAVI, transcatheter aortic valve implantation; VHD, valvular heart disease.

Among patients hospitalized for a first episode of AHF, younger age, male sex, absence of MI, admission to a university hospital, and reduced LVEF were independently associated with CMR examination (see Supplementary data online, *Table S6*, *Figure 3B*).

### CMR rates across hospital types and wards

**Figure 4:**
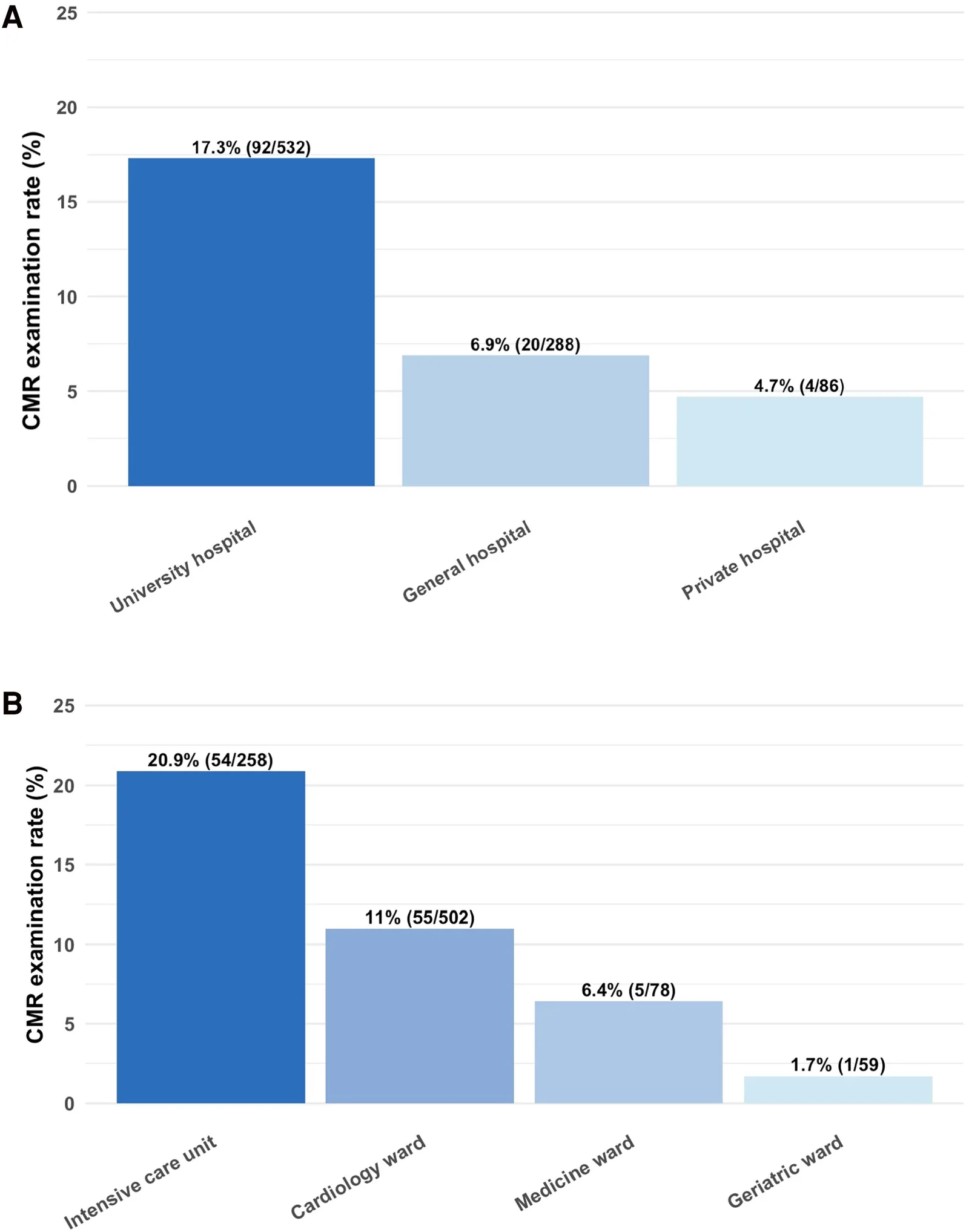
Rates of CMR according to hospital type (*A*) and admission ward (*B*).

CMR use varied markedly by hospital type. University hospitals showed the highest rates (*n* = 92/532, 17.3%), compared with general (*n* = 20/288, 6.9%) and private hospitals (*n* = 4/86, 4.7%, *P* < 0.001, *Figure 4A*). CMR rates also differed across hospital wards: the highest were in ICU/ICCU (n = 54/258, 20.9%), intermediate in cardiology wards (*n* = 55/502, 11.0%), and lowest in medicine (*n* = 5/78, 6.4%) and geriatric units (*n* = 1/59, 1.7%, *P* < 0.001, *Figure 4B*).

### Adherence to ESC guideline-based CMR indications

CMR use was then assessed relative to Class I and Class II indications from the 2016 ESC Guidelines for the Diagnosis and Treatment of Acute and Chronic Heart Failure, which were in effect during the study period. Overall, only a minority of eligible patients underwent CMR (*Table 3*). Among those with a Class I indication (*n* = 46), 6.5% had a CMR in the 2 years before admission and 10.9% during hospitalization or within 1 year after discharge. For Class II indications (*n* = 167), these proportions were 5.4% and 15.0%, respectively.

**Table 3:** Adherence of CMR utilization according to ESC guidelines

Recommendation	Total number of patients	CMR within 2 years before index hospitalization	CMR during or within 1 year after index hospitalization
**ESC 2016—Class I**	46	6.5%	10.9%
**ESC 2016—Class II**	167	5.4%	15.0%

## Discussion

Based on data from the national, prospective, multicentre OFICA2 cohort of patients hospitalized for AHF, we examined real-world use of CMR and its associations with demographic, clinical, and institutional characteristics. The main findings were as follows. The overall CMR use was low, including among patients with a first diagnosis of HF. CMR was more frequently performed in younger individuals, without MI, with reduced LVEF, and presenting dilated rather than valvular or hypertensive cardiomyopathy. Referral was also more common in university hospitals and intensive care units. In patients hospitalized for a first episode of AHF, male sex was additionally associated with CMR examination. Finally, concordance between CMR use and guideline-based indications was low, highlighting a considerable gap between recommendations and clinical practice.

### Low utilization of CMR in patients hospitalized for AHF

The low CMR examination rate observed in our study is consistent with international data and appears to reflect structural and logistical barriers.^10–12^ In the EORP Cardiomyopathy/Myocarditis Registry, CMR was performed in 29.4% of patients, almost twice the rate observed here.^13^ Notably, EORP enrolled patients with a class I recommendation for CMR in centres with cardiomyopathy expertise, whereas OFICA2 included all cardiac diseases across all hospital types, providing a more representative overview of AHF management. Surveys consistently identify restricted CMR access as the main limitation, with delays incompatible with timely decision-making.^11^ It is also likely that not all physicians have fully integrated the value of CMR into their routine clinical practice, leading to examination rates that remain low.

### Clinical and demographic factors associated with CMR examination

Older patients were less referred for CMR, likely reflecting greater comorbidity, frailty, and perceived limited clinical benefit. Yet this underuse risks missed diagnoses. HFpEF was also independently associated with lower CMR use, despite its established value in identifying specific underlying aetiologies across the HFpEF spectrum, including amyloidosis.^14^

Similarly, MI was associated with lower CMR referral. In practice, CMR is not routinely incorporated into acute MI management and is mainly reserved for cases of inconclusive echocardiography, suspected left ventricular thrombus, MI with non-obstructive coronary arteries, or later myocardial viability.^15^

Patients with severe valvular heart disease were also less likely to undergo CMR, as echocardiography often suffices for diagnosis and decision-making. In aortic stenosis, CMR is not routinely required, although mid-wall fibrosis carries prognostic value.^16^ In valvular regurgitation, CMR is mainly useful when echocardiographic quantification remains uncertain, although advanced techniques such as 4D-flow, while promising for directly assessing regurgitant volumes, are not widely available in routine practice.^17^

Conversely, patients with an initial diagnosis of dilated cardiomyopathy were more frequently referred, consistent with robust evidence supporting the central role of CMR in evaluating these patients.^3^

Finally, in patients with a first episode of AHF, male sex was associated with higher CMR examination rates. Women were on average 5 years older than men, which may have contributed to a lower likelihood of CMR use in routine practice. However, male sex remained independently associated with CMR use after multivariable adjustment that included age. Beyond age, no major differences in baseline characteristics were identified that could sufficiently explain the observed association. Therefore, the mechanisms underlying this sex-related difference cannot be determined from our observational data, and unequal access to advanced imaging or differences in referral patterns cannot be excluded. A subconscious referral bias has been documented in women with ischaemic heart disease, and sex-related underutilization has been reported for other advanced cardiac diagnostic modalities.^18,19^ Finally, residual confounding cannot be ruled out in this observational study.

It is noteworthy that data specifically addressing sex-related disparities in real-world CMR use remain scarce. To the best of our knowledge, no published dedicated study has addressed this finding. A large TriNetX database analysis, currently available only as a conference abstract, reported lower CMR use among women across several cardiovascular conditions, including heart failure.^20^ Conversely, in the EORP Cardiomyopathy/Myocarditis Registry, sex was not identified among the determinants of CMR use.^13^ These limited and partly conflicting findings highlight the need for dedicated studies investigating sex-related disparities in referral for and access to CMR.^21,22^

### Institutional disparities in CMR examination rates

CMR was more often performed in university hospitals, as in other countries.^12^ This likely reflects the concentration of expertise and presence of dedicated CMR platforms within tertiary hospitals. Conversely, some general or private centres face limited access to trained staff, with probable lower CMR examination rates, restricted scanner availability, and a financial valuation of CMR that remains insufficient compared with the time and expertise required.^23^ Together, these factors reinforce the perception of CMR as an ‘expert-level’ test rather than a routine tool integrated into everyday HF care.

### Adherence of CMR use to European guidelines

Only a small proportion of eligible patients received CMR according to the 2016 ESC Heart Failure guidelines (10.9% class I, 15.0% class II), which were the reference guidelines at the time of patient inclusion, confirming a major gap between recommendations and real-world practice. This gap is even more relevant today, as recent ESC updates have further expanded the role of CMR, particularly in cardiomyopathies.^5,24^

### Clinical perspectives

The findings of our study highlight several priorities to better integrate CMR into routine HF care.

As in other countries, expanding access to CMR requires sufficiently trained specialists. Current challenges in meeting healthcare demands reflect not only the rising volume of imaging requests across all medical fields but also the central role that radiologists and cardiologists play in ensuring high-quality procedures and safe interpretation of these examinations. Scientific societies and local institutions should support inter-specialty, competency-based training during fellowship to strengthen collaboration and optimize patient care, while respecting the agreements and organizational frameworks already established between specialties.^23^ Review of patient data by a cardiologist before acquisition can help tailor protocols and optimize diagnostic yield.^25,26^ Training of MRI technologists is equally essential, as cardiac imaging requires knowledge of cardiac anatomy and real-time coordination with the medical team during acquisition.

Reducing examination time is also key. A standard scan still lasts about 40 min, limiting its feasibility in busy settings and among less-tolerant patients. Pragmatic, pathology-driven protocols should aim for 30-minute examinations.^25,27^ Future developments, including artificial intelligence, are expected to improve CMR workflow and accessibility.^28^ However, efforts should also ensure that technological advances developed in research settings translate into routine clinical practice rather than widening disparities between centres. Improving access to CMR will also require appropriate financial valuation of the examination and expansion of MRI capacity. Finally, these developments should be accompanied by greater attention to the environmental sustainability of CMR.^29^

### Study limitations

OFICA2 is a prospective, multicentre cohort of patients hospitalized for AHF in France and may not reflect HF management elsewhere, particularly in low-income countries. The study population included only hospitalized patients, thereby excluding those managed in outpatient settings.

We did not have detailed information on specific contraindications to CMR such as claustrophobia or non-retrievable piercings, and the results may thus be underestimated. However, given the very low overall rate of CMR use, these factors are unlikely to materially affect our findings. Claustrophobia is relatively uncommon, and body piercings can usually be manageable before MRI.^30^

We assessed the rate of CMR examinations rather than actual CMR prescriptions, as prescription data were not available. It is likely that some patients were never prescribed a CMR, owing to limited awareness or adherence to recent guidelines.

For the analysis of adherence to ESC recommendations, information on some specific aetiologies such as cardiac sarcoidosis, Chagas disease, Fabry disease, or hemochromatosis was not available in the registry; however, these conditions remain rare in the context of AHF.

For the primary endpoint, the 1-year follow-up window was intended to capture CMR examinations deferred until clinical stabilization and outpatient reassessment. We cannot exclude the possibility that some examinations performed several months after discharge reflected a different clinical context.

Finally, this study was not designed to determine whether CMR modified downstream management or outcomes but rather to quantify real-world use of CMR after hospitalization for AHF in a broad national cohort.

## Conclusion

In this contemporary nationwide cohort of patients hospitalized for AHF, real-world use of CMR remained limited, even among patients with potential guideline-based indications. CMR was more often used in younger patients, in those without MI, in those with reduced LVEF, and in cases of dilated rather than valvular or hypertensive cardiomyopathy; among patients hospitalized for a first episode of AHF, male sex was also associated with referral. Marked differences in CMR use were also observed across hospital types. These findings call for coordinated initiatives between cardiology and radiology to support the broader integration of CMR into routine heart failure care.

## Supplementary Material

qyag159_Supplementary_Data

## Data Availability

The data that support the findings of this study are available from the corresponding author upon reasonable request.

## Lead author biography

Dr Alexandre Altes is a cardiologist specializing in cardiac imaging, including echocardiography and cardiovascular magnetic resonance, and an Associate Professor of Cardiology at Lille Catholic University, France. He is a member of the DataCoeur research team, ETHICS EA7446. His research focuses on personalized medicine and refined prognostic stratification in native valvular heart disease through multimodality imaging. He completed his PhD under the supervision of Professor Sylvestre Maréchaux and is a former fellow at Cliniques Universitaires Saint-Luc, Brussels, under the supervision of Professor Bernhard L. Gerber. EACVI Level 3-certified in cardiovascular magnetic resonance and a Fellow of the European Society of Cardiology, he is also a member of the Research Committee of the French Society of Cardiology. His work aims to improve the precision and prognostic value of cardiovascular imaging in clinical practice.
