# Supplementary material for: Use of cardiac magnetic resonance imaging after hospitalization for acute heart failure: insights from the French nationwide OFICA-2 cohort
Source: Eur Heart J Imaging Methods Pract. 2026 Sep 10;4(3):qyag159. doi: 10.1093/ehjimp/qyag159 (PMC13592046; doi:10.1093/ehjimp/qyag159)
Supplement: Supplementary file 1 [file qyag159_supplementary_data.docx]

| **Variables** | | | **Patients with CMR**  **N = 147** | | | | **Patients with available data, n** | |
| --- | --- | --- | --- | --- | --- | --- | --- | --- |
| **Demographics** | | |  | | |  | | |
| Age (years) | | 62.7 ± 15.3 | | | 147 | | |  |
| Male gender, n (%) | | 106 (72.1%) | | | 147 | | |  |
| Hypertension, n (%) | | 77 (52.4%) | | | 147 | | |  |
| Active smoker, n (%) | | 38 (25.2%) | | | 147 | | |  |
| Diabetes mellitus, n (%) | | 41 (28.1%) | | | 146 | | |  |
| SBP (mmHg) | | 135.6 ± 30.2 | | | 144 | | |  |
| Heart Rate (b.p.m.) | | 96.6 ± 27.0 | | | 145 | | |  |
| Sinus rhythm | | 94 (64.4%) | | | 146 | | |  |
| BMI (kg/m²) | | 27.6 ± 6.4 | | | 133 | | |  |
| Obesity | | 32 (24.1%) | | | 133 | | |  |
| **Cardiovascular history** | |  | | |  | | |  |
| Past HF hospitalization | | 50 (34.2%) | | | 146 | | |  |
| MI | | 22 (15.0%) | | | 147 | | |  |
| PCI | | 18 (12.2%) | | | 147 | | |  |
| CABG | | 7 (4.8%) | | | 147 | | |  |
| Valvular surgery | | 3 (2.0%) | | | 147 | | |  |
| TAVI | | 0 (0%) | | | 146 | | |  |
| Cerebrovascular disease | | 6 (4.1%) | | | 147 | | |  |
| **Cardiovascular history** | |  | | |  | | |  |
| Alcohol abuse | | 25 (17.0%) | | | 147 | | |  |
| Treated obstructive sleep apnea | | 13 (8.8%) | | | 147 | | |  |
| Treated COPD | | 28 (19.2%) | | | 146 | | |  |
| Treated depression | | 9 (6.2%) | | | 146 | | |  |
| Cirrhosis | | 5 (3.4%) | | | 147 | | |  |
| Active or recent (< 5 years) cancer | | 12 (8.2%) | | | 147 | | |  |
| Severe cognitive impairment | | 1 (0.7%) | | | 146 | | |  |
| Charlson comorbidity index | | 3.5 ± 3.0 | | | 147 | | |  |
| **Index hospitalization** |  | | |  | | | | |
| Type of hospital | |  | | | 147 | | |  |
| - University hospital | | 112 (76.2%) | | |  | | |  |
| - General hospital | | 31 (21.1%) | | |  | | |  |
| - Private hospital | | 4 (2.7%) | | |  | | |  |
| Hospitalization wards | |  | | | 147 | | |  |
| - Cardiology wards | | 76 (51.7%) | | |  | | |  |
| - ICCU/ICU | | 61 (41.5%) | | |  | | |  |
| - Geriatry wards | | 1 (0.7%) | | |  | | |  |
| - Medicine wards | | 8 (5.4%) | | |  | | |  |
| - Other | | 0 (0%) | | |  | | |  |
| Hemoglobin (g/dl) | | 13.0 ± 2.3 | | | 143 | | |  |
| eGFR (ml/min/1.73m²) | | 75.2 ± 24.2 | | | 140 | | |  |

**Supplemental Table 1. Baseline clinical and demographic characteristics of patients who underwent CMR either during the index hospitalization for AHF, within the two years preceding it, or within one year following discharge**

Quantitative data are expressed as mean ± SD or median IQR according to distribution and qualitative data as count (%).

BMI, Body Mass Index; CABG, Coronary Artery Bypass Grafting; COPD, Chronic Obstructive Pulmonary Disease; eGFR, estimated Glomerular Filtration Rate; HF, Heart Failure; ICCU, Intensive Cardiac Care Unit; ICU, Intensive Care Unit; MI, Myocardial Infarction; PCI, Percutaneous Coronary Intervention; SBP, Systolic Blood Pressure; TAVI, Transcatheter Aortic Valve Implantation.

| **Variables** | | **Patients with CMR**  **N = 147** | | | **Patients with available data, n** | |
| --- | --- | --- | --- | --- | --- | --- |
| **Etiology** | |  | | 147 | | |
| VHD | 3 (2.0%) | |  | | |  |
| Ischemic | 47 (32.0%) | |  | | |  |
| DCM | 55 (37.4%) | |  | | |  |
| Arrythmia-induced | 13 (8.8%) | |  | | |  |
| Myocarditis | 3 (2.0%) | |  | | |  |
| Hypertension | 1 (0.7%) | |  | | |  |
| Other | 19 (12.9%) | |  | | |  |
| **LVEF category** |  | | 147 | | |  |
| Preserved LVEF | 34 (23.1%) | |  | | |  |
| Mid-range LVEF | 11 (7.5%) | |  | | |  |
| Altered LVEF | 100 (68.0%) | |  | | |  |
| **Type of HF** |  | | 147 | | |  |
| Right-sided | 44 (29.9%) | |  | | |  |
| Left-sided | 71 (48.3%) | |  | | |  |
| Global | 30 (20.4%) | |  | | |  |

**Supplemental Table 2. Etiology of cardiopathy and LVEF categories of patients who underwent CMR either during the index hospitalization for AHF, within the two years preceding it, or within one year following discharge**

DCM; Dilated Cardiomyopathy; HF: Heart Failure; LVEF: Left Ventricular Ejection Fraction; VHD: Valvular Heart Disease

| **Variables** | | | **Patients hospitalized for a first episode of AHF**  **N = 461** | | | | **Women**  **N = 205**  **(44.5%)** | | | | **Men**  **N = 256 (55.5%)** | | | | **P-value** | | | | **Patients with available data, n** | |
| --- | --- | --- | --- | --- | --- | --- | --- | --- | --- | --- | --- | --- | --- | --- | --- | --- | --- | --- | --- | --- |
| CMR during hospitalization or within one year, n (%) | | | 76 (16.5%) | | | 24 (11.7%) | | | | 52 (20.3%) | | | | **0.019** | | | | 461 | | |
| **Demographics** | | |  | | |  | | | |  | | | |  | | | |  | | |
| Age (years) | | 73.1 ± 14.4 | | | 75.9 ± 14.6 | | | | 70.9 ± 13.9 | | | | **<0.001** | | | | 461 | | |  |
| Hypertension, n (%) | | 297 (65.0%) | | | 132 (65.0%) | | | | 165 (65.0%) | | | | >0.99 | | | | 457 | | |  |
| Active smoker, n (%) | | 73 (16.0%) | | | 22 (10.9%) | | | | 51 (20.0%) | | | | **0.013** | | | | 456 | | |  |
| Diabetes mellitus, n (%) | | 152 (33.4%) | | | 58 (28.7%) | | | | 94 (37.2%) | | | | 0.072 | | | | 455 | | |  |
| SBP (mmHg) | | 138.2 ± 30.1 | | | 139.1 ± 30.0 | | | | 137.5 ± 30.1 | | | | 0.555 | | | | 453 | | |  |
| Heart Rate (b.p.m.) | | 96.3 ± 27.0 | | | 100.9 ± 28.8 | | | | 92.5 ± 24.8 | | | | **0.002** | | | | 455 | | |  |
| Sinus rhythm | | 261 (57.2%) | | | 115 (56.1%) | | | | 146 (58.2%) | | | | 0.727 | | | | 456 | | |  |
| BMI (kg/m²) | | 29.0 ± 7.6 | | | 29.2 ± 8.6 | | | | 28.8 ± 6.8 | | | | 0.684 | | | | 397 | | |  |
| Obesity | | 144 (36.3%) | | | 69 (40.1%) | | | | 75 (33.3%) | | | | 0.198 | | | | 397 | | |  |
| **Cardiovascular history** | |  | | |  | | | |  | | | |  | | | |  | | |  |
| Past HF hospitalization | | 3 (0.7%) | | | 1 (0.5%) | | | | 2 (0.8%) | | | | >0.99 | | | | 460 | | |  |
| MI | | 54 (11.7%) | | | 15 (7.3%) | | | | 39 (15.2%) | | | | **0.013** | | | | 461 | | |  |
| PCI | | 53 (11.5%) | | | 13 (6.4%) | | | | 40 (15.6%) | | | | **0.003** | | | | 460 | | |  |
| CABG | | 15 (3.3%) | | | 2 (1.0%) | | | | 13 (5.1%) | | | | **0.028** | | | | 460 | | |  |
| Valvular surgery | | 16 (3.5%) | | | 6 (2.9%) | | | | 10 (3.9%) | | | | 0.760 | | | | 460 | | |  |
| TAVI | | 8 (1.7%) | | | 6 (2.9%) | | | | 2 (0.8%) | | | | 0.147 | | | | 460 | | |  |
| Cerebrovascular disease | | 34 (7.4%) | | | 15 (7.4%) | | | | 19 (7.4%) | | | | >0.99 | | | | 459 | | |  |
| **Non cardiovascular history** | |  | | |  | | | |  | | | |  | | | |  | | |  |
| Alcohol abuse | | 54 (11.8%) | | | 10 (5.0%) | | | | 44 (17.3%) | | | | **<0.001** | | | | 457 | | |  |
| Treated obstructive sleep apnea | | 31 (6.8%) | | | 5 (2.5%) | | | | 26 (10.2%) | | | | **0.002** | | | | 458 | | |  |
| Treated COPD | | 64 (14.0%) | | | 22 (10.9%) | | | | 42 (16.5%) | | | | 0.116 | | | | 457 | | |  |
| Treated depression | | 38 (8.3%) | | | 25 (12.3%) | | | | 13 (5.1%) | | | | **0.009** | | | | 458 | | |  |
| Cirrhosis | | 14 (3.1%) | | | 2 (1.0%) | | | | 12 (4.7%) | | | | **0.042** | | | | 455 | | |  |
| Active or recent (< 5 years) cancer | | 32 (7.0%) | | | 14 (7.0%) | | | | 18 (7.1%) | | | | >0.99 | | | | 455 | | |  |
| Severe cognitive impairment | | 13 (2.9%) | | | 8 (4.0%) | | | | 5 (2.0%) | | | | 0.324 | | | | 456 | | |  |
| Charlson comorbidity index | | 3.3 ± 3.0 | | | 3.3 ± 3.1 | | | | 3.4 ± 2.9 | | | | 0.266 | | | | 461 | | |  |
| **Index hospitalization** |  | | |  | | | |  | | | |  | | | |  | | | | |
| Type of hospital | |  | | |  | | | |  | | | | 0.731 | | | | 461 | | |  |
| - University hospital | | 275 (59.7%) | | | 126 (61.5%) | | | | 149 (58.2%) | | | |  | | | |  | | |  |
| - General hospital | | 139 (30.2%) | | | 58 (28.3%) | | | | 81 (31.6%) | | | |  | | | |  | | |  |
| - Private hospital | | 47 (10.2%) | | | 21 (10.2%) | | | | 26 (10.2%) | | | |  | | | |  | | |  |
| Hospitalization wards | |  | | |  | | | |  | | | | 0.440 | | | | 461 | | |  |
| - Cardiology wards | | 239 (51.8%) | | | 107 (52.2%) | | | | 132 (51.6%) | | | |  | | | |  | | |  |
| - ICCU/ICU | | 161 (34.9%) | | | 72 (35.1%) | | | | 89 (34.8%) | | | |  | | | |  | | |  |
| - Geriatry wards | | 19 (4.1%) | | | 11 (5.4%) | | | | 8 (3.1%) | | | |  | | | |  | | |  |
| - Medicine wards | | 37 (8.0%) | | | 13 (6.3%) | | | | 24 (9.4%) | | | |  | | | |  | | |  |
| - Other | | 0 (0%) | | | 0 (0%) | | | | 0 (0%) | | | |  | | | |  | | |  |
| Hemoglobin (g/dl) | | 12.8 ± 2.3 | | | 12.4 ± 2.3 | | | | 13.1 ± 2.3 | | | | **0.001** | | | | 447 | | |  |
| eGFR (ml/min/1.73m²) | | 68.9 ± 22.2 | | | 66.5 ± 21.7 | | | | 70.8 ± 22.5 | | | | **0.045** | | | | 447 | | |  |

**Supplemental Table 3. Baseline clinical and demographic characteristics of patients hospitalized for a first episode of acute heart failure, stratified by sex**

BMI, Body Mass Index; CABG, Coronary Artery Bypass Grafting; COPD, Chronic Obstructive Pulmonary Disease; eGFR, estimated Glomerular Filtration Rate; HF, Heart Failure; ICCU, Intensive Cardiac Care Unit; ICU, Intensive Care Unit; MI, Myocardial Infarction; PCI, Percutaneous Coronary Intervention; SBP, Systolic Blood Pressure; TAVI, Transcatheter Aortic Valve Implantation.

| **Variables** | | **Patients hospitalized for a first episode of AHF**  **N = 461** | | | **Women**  **N = 205**  **(44.5%)** | | | **Men**  **N = 256 (55.5%)** | | | **P-value** | | | **Patients with available data, n** | |
| --- | --- | --- | --- | --- | --- | --- | --- | --- | --- | --- | --- | --- | --- | --- | --- |
| **Etiology** | |  | |  | | |  | | | **0.007** | | | 451 | | |
| - VHD | 47 (10.2%) | | 19 (9.3%) | | | 28 (10.9%) | | |  | | |  | | |  |
| - Ischemic | 126 (27.3%) | | 48 (23.4%) | | | 78 (30.5%) | | |  | | |  | | |  |
| - DCM | 84 (18.2%) | | 34 (16.6%) | | | 50 (19.5%) | | |  | | |  | | |  |
| - Arrythmia-induced | 95 (20.6%) | | 56 (27.3%) | | | 39 (15.2%) | | |  | | |  | | |  |
| - Myocarditis | 3 (0.7%) | | 0 (0%) | | | 3 (1.2%) | | |  | | |  | | |  |
| - Hypertension | 23 (5.0%) | | 14 (6.8%) | | | 9 (3.5%) | | |  | | |  | | |  |
| - Other | 73 (15.8%) | | 28 (13.7%) | | | 45 (17.6%) | | |  | | |  | | |  |
| **LVEF category** |  | |  | | |  | | | 0.276 | | | 435 | | |  |
| Preserved LVEF | 190 (41.2%) | | 92 (44.9%) | | | 98 (38.3%) | | |  | | |  | | |  |
| Mid-range LVEF | 46 (10.0%) | | 18 (8.8%) | | | 28 (10.9%) | | |  | | |  | | |  |
| Altered LVEF | 199 (43.2%) | | 82 (40.0%) | | | 117 (45.7%) | | |  | | |  | | |  |
| **Type of HF** |  | |  | | |  | | | **0.020** | | | 454 | | |  |
| Right-sided | 145 (31.5%) | | 51 (24.9%) | | | 94 (36.7%) | | |  | | |  | | |  |
| Left-sided | 200 (43.4%) | | 99 (48.3%) | | | 101 (39.5%) | | |  | | |  | | |  |
| Global | 109 (23.6%) | | 53 (25.9%) | | | 56 (21.9%) | | |  | | |  | | |  |

**Supplemental Table 4. Etiology of cardiopathy and LVEF categories of patients hospitalized for a first episode of acute heart failure, stratified by sex**

DCM; Dilated Cardiomyopathy; HF: Heart Failure; LVEF: Left Ventricular Ejection Fraction; VHD: Valvular Heart Disease

| **Variables** | | **Patients eligible to CMR**  **N = 906** | | **Patients**  **with CMR**  **N = 116 (12.8%)** | | **Patients**  **without CMR**  **N = 790 (87.2%)** | **Overall**  **p-value** | **Patients with available data, n** |
| --- | --- | --- | --- | --- | --- | --- | --- | --- |
| **Etiology** | | |  | |  |  |  | 881 |
| - VHD | | | 118 (13.0%) | | 2 (1.7%) | 116 (14.7%) | **<0.001** |  |
| - Ischemic | | | 257 (28.4%) | | 35 (30.2%) | 222 (28.1%) | 0.725 |  |
| - DCM | | | 153 (16.9%) | | 48 (41.4%) | 105 (13.3%) | **<0.001** |  |
| - Arrythmia-induced | | | 172 (19.0%) | | 8 (6.9%) | 164 (20.8%) | **<0.001** |  |
| - Myocarditis | | | 4 (0.4%) | | 3 (2.6%) | 1 (0.1%) | **0.003** |  |
| - Hypertension | | | 39 (4.3%) | | 1 (0.9%) | 38 (4.8%) | 0.087 |  |
| - Other | | | 138 (15.2%) | | 14 (12.1%) | 124 (15.7%) | 0.380 |  |
| **LVEF category** | | |  | |  |  |  | 860 |
| - Preserved LVEF | | | 382 (42.2%) | | 22 (19.0%) | 360 (45.6%) | **<0.001** |  |
| - Mid-range LVEF | | | 100 (11.0%) | | 10 (8.6%) | 90 (11.4%) | 0.465 |  |
| - Altered LVEF | 378 (41.7%) | | | | 83 (71.6%) | 295 (37.3%) | **<0.001** |  |
| **Type of HF** |  | | | |  |  |  | 895 |
| - Right-sided | 323 (35.7%) | | | | 34 (29.3%) | 289 (36.6%) | 0.155 |  |
| - Left-sided | 345 (38.1%) | | | | 56 (48.3%) | 289 (36.6%) | **0.020** |  |
| - Global | 227 (25.1%) | | | | 24 (20.7%) | 203 (25.7%) | 0.295 |  |

Qualitative data are expressed as count (%).

DCM; Dilated Cardiomyopathy; HF: Heart Failure; LVEF: Left Ventricular Ejection Fraction; VHD: Valvular Heart Disease

**Supplemental Table 5. Etiology of cardiopathy and LVEF categories by CMR prescription**

|  | **Univariate analysis** | | | | **Multivariable analysis (n=447)** | | |
| --- | --- | --- | --- | --- | --- | --- | --- |
| **Covariates** | | **OR (95% CI)** | **P value** | **Patients**  **with available data, n** | | **OR (95% CI)** | **P value** |
| Male gender | | 1.92 (1.14 – 3.24) | 0.014 | 461 | | 2.12 (1.09 – 4.12) | **0.026** |
| Age (year) | | 0.93 (0.91 – 0.95) | <0.001 | 461 | | 0.94 (0.92 – 0.96) | **<0.001** |
| Hypertension | | 0.30 (0.18 – 0.49) | <0.001 | 457 | |  |  |
| Active smoker | | 2.63 (1.48 – 4.68) | 0.001 | 456 | |  |  |
| Heart rate (b.p.m) | | 1.01 (1.00 – 1.02) | 0.139 | 461 | |  |  |
| Obesity | | 0.48 (0.26 – 0.88) | 0.018 | 397 | |  |  |
| MI | | 0.48 (0.19 – 1.26) | 0.135 | 461 | | 0.30 (0.10 – 0.94) | **0.039** |
| PCI | | 0.27 (0.08 – 0.90) | 0.034 | 460 | |  |  |
| Valvular surgery | | 0.33 (0.04 – 2.52) | 0.284 | 460 | |  |  |
| Cerebrovascular disease | | 0.30 (0.07 – 1.26) | 0.100 | 459 | |  |  |
| Alcohol abuse | | 1.93 (0.99 – 3.75) | 0.054 | 457 | |  |  |
| Severe cognitive impairment | | 0.42 (0.05 – 3.24) | 0.402 | 456 | |  |  |
| Hemoglobin (g/dl) | | 1.17 (1.04 – 1.31) | 0.008 | 447 | |  |  |
| eGFR (ml/min/1.73m²) | | 0.99 (0.99 – 1.00) | 0.248 | 447 | | 0.99 (0.98 – 1.00) | 0.240 |
| University hospital | | 3.39 (1.72 – 6.67) | <0.001 | 461 | | 2.93 (1.43 – 6.02) | **0.003** |
| Private hospital | | 0.79 (0.21 – 2.98) | 0.731 | 461 | |  |  |
| Cardiology wards | | 0.67 (0.41 – 1.09) | 0.109 | 461 | | 1.07 (0.59 – 1.95) | 0.820 |
| ICCU/ICU | | 2.27 (1.38 – 3.74) | 0.001 | 461 | |  |  |
| DCM | | 7.59 (2.96 – 19.48) | <0.001 | 451 | |  |  |
| Arrhythmia-related | | 0.62 (0.18 – 2.12) | 0.446 | 451 | |  |  |
| Hypertensive | | 0.51 (0.06 – 4.45) | 0.540 | 451 | |  |  |
| Ischemic | | 2.76 (1.08 – 7.10) | 0.035 | 451 | | 1.86 (0.93 – 3.69) | 0.078 |
| Myocarditis | | 1.60E10 (0.00 – Inf) | 0.999 | 451 | |  |  |
| Severe VHD | | 0.24 (0.03 – 2.08) | 0.197 | 451 | |  |  |
| Reduced LVEF (<40%) | | 6.07 (3.41 – 10.83) | <0.001 | 461 | |  |  |
| Preserved LVEF (>50%) | | 0.17 (0.09 – 0.35) | <0.001 | 461 | | 0.29 (0.14 – 0.63) | **0.002** |
| Left-sided HF | | 1.67 (1.02 – 2.73) | 0.043 | 461 | |  |  |

**Supplemental Table 6. Factors associated with CMR use in patients hospitalized for a first episode of acute heart failure on univariate and multivariable analysis**

BMI, Body Mass Index; CABG, Coronary Artery Bypass Grafting; COPD, Chronic Obstructive Pulmonary Disease; DCM, Dilated Cardiomyopathy; eGFR, estimated Glomerular Filtration Rate; HF, Heart Failure; ICCU, Intensive Cardiac Care Unit; ICU, Intensive Care Unit; MI, Myocardial Infarction; PCI, Percutaneous Coronary Intervention; SBP, Systolic Blood Pressure; TAVI, Transcatheter Aortic Valve Implantation; VHD, Valvular Heart Disease
